# The Use of UV Spectroscopy and SIMCA for the Authentication of Indonesian Honeys According to Botanical, Entomological and Geographical Origins

**DOI:** 10.3390/molecules26040915

**Published:** 2021-02-09

**Authors:** Diding Suhandy, Meinilwita Yulia

**Affiliations:** 1 Department of Agricultural Engineering, Faculty of Agriculture, The University of Lampung, Jl. Prof. Dr. Soemantri Brojonegoro No.1, Bandar Lampung 35145, Indonesia; 2 Department of Agricultural Technology, Lampung State Polytechnic, Jl. Soekarno Hatta No. 10, Rajabasa, Bandar Lampung 35141, Indonesia; meinilwitayulia@polinela.ac.id

**Keywords:** UV spectroscopy, authentication, botanical origin, geographical origin, Indonesian honey, entomological origin

## Abstract

As a functional food, honey is a food product that is exposed to the risk of food fraud. To mitigate this, the establishment of an authentication system for honey is very important in order to protect both producers and consumers from possible economic losses. This research presents a simple analytical method for the authentication and classification of Indonesian honeys according to their botanical, entomological, and geographical origins using ultraviolet (UV) spectroscopy and SIMCA (soft independent modeling of class analogy). The spectral data of a total of 1040 samples, representing six types of Indonesian honey of different botanical, entomological, and geographical origins, were acquired using a benchtop UV-visible spectrometer (190–400 nm). Three different pre-processing algorithms were simultaneously evaluated; namely an 11-point moving average smoothing, mean normalization, and Savitzky–Golay first derivative with 11 points and second-order polynomial fitting (ordo 2), in order to improve the original spectral data. Chemometrics methods, including exploratory analysis of PCA and SIMCA classification method, was used to classify the honey samples. A clear separation of the six different Indonesian honeys, based on botanical, entomological, and geographical origins, was obtained using PCA calculated from pre-processed spectra from 250–400 nm. The SIMCA classification method provided satisfactory results in classifying honey samples according to their botanical, entomological, and geographical origins and achieved 100% accuracy, sensitivity, and specificity. Several wavelengths were identified (266, 270, 280, 290, 300, 335, and 360 nm) as the most sensitive for discriminating between the different Indonesian honey samples.

## 1. Introduction

According to the Codex Alimentarius Commission [1], honey is defined as “the natural sweet substance produced by honey bees from the nectar of plants or from secretions of living parts of plants, which the bees collect, transform by combining with specific substances of their own, deposit, dehydrate, store and leave in the honey comb to ripen and mature”. The main components of honey are carbohydrates (e.g., glucose and fructose in nearly 75% *w/w*), organic acids, amino acids, vitamins, volatile oils, and minerals [2,3,4]. Moreover, its minor components and appearance are highly affected by different sources of nectar (botanical origin), different types of honeybees, and geographical factors, such as beekeeping practices, climate, and storage conditions [5]. In terms of botanical origin, honey can be categorized into two broad types, namely monofloral and multifloral honey. Generally, due to limited production and availability, monofloral honey is more valued and consequently has a higher market price than multifloral honey [3,6]. Currently, Indonesian beekeepers use several species of honeybee, including *Apis dorsata*, *Apis mellifera* and *Apis cerana L*. *Apis dorsata* honey, in general, is more expensive than *Apis mellifera* honey due to its infrequent production and massive deforestation. The main area of production is the island of Sumbawa, which accounts for almost 80% of total national honey production in Indonesia.

Honey is regarded as a functional food with health-promoting and disease-preventing properties, and is typically high in important nutrients [7]. For this reason, honey’s popularity and high market value expose it to the risk of food fraud. According to Valand et al. [8], food fraud is a collective term that encompasses the deliberate substitution, addition, tampering or misrepresentation of food, food ingredients or food packaging, or false or misleading statements made about a product for economic gain. Fraudulent practices involved in honey production and sales include adulteration of honey with sugar or syrup and mislabeling of botanical, entomological, and geographical origin [9]. To ensure fair trading of such high valued products as honey, an authentication system needs to be established to detect fraud in the honey supply chain. Most reported works on honey authentication mainly focus on the floral type (botanical origin) and geographical origins, and less frequently on entomological origins [10,11,12,13,14]. Few reported works have been reported on the honey authentication based on entomological origins [15,16].

To date, several authentication protocols for the natural product including honey using various analytical methods implemented with chemometrics have been reported [3,17,18,19]. According to Chin and Sowndhararajan [20], there are two main analytical methods used in honey classification, identification, and authentication: classical and modern methods. Two popular classical methods are physicochemical analysis for determining monofloral botanical honey origins from Ortigueira, Brazil [21], and a melissopalynological approach for identifying floral pollen grains present in Egyptian honey [22]. While these two classical methods are accurate, they are time consuming, tedious to implement, and require highly trained personnel to perform them. Included among the modern methods available for honey authentication are chromatographic, mass spectrometry, spectroscopy (UV-visible, near infrared (NIR), mid infrared (MIR) and terahertz (THz)), nuclear magnetic resonance/NMR (^1^H NMR, ^13^C NMR), and molecular (real-time PCR) techniques [20]. Both chromatographic and mass spectrometry methods are time consuming and involve expensive devices and laborious sample preparation. While spectroscopy in the NIR, mid-infrared and THz regions has acceptable accuracy, is quite fast, and needs little sample preparation, it does require expensive instruments. NMR is also accurate, but it is time consuming and uses expensive equipment. Recently, a real-time polymerase chain reaction (PCR) procedure was used to detect Spanish honey adulteration [23]. It was concluded that real-time PCR could detect adulteration of honey with rice molasses at very low levels of adulteration. However, this technique is quite expensive, time consuming, and requires laborious data acquisition.

Recently, UV spectroscopy for food analysis has received increasing attention due to multiple advantages, e.g., being simple, relatively fast, requiring little or no sample preparation, and the use of relatively inexpensive equipment [24,25]. UV spectroscopy utilizes the wavelength range from 200–400 nm and has been used for the authentication of expensive Sidr Yemeni honey with acceptable results [26] and was further validated by Ansari et al. [7]. The UV model developed according to Roshan et al. [26] successfully differentiated the botanical source of Saudi honey samples properly. This UV method is relatively fast, inexpensive, and easy to implement for routine analysis. However, their reported methodology includes the use of a chemical solvent (ethanol) for sample preparation. Previously, Suhandy and co-workers applied low-cost UV-visible spectroscopy for the detection of adulteration of Indonesian specialty coffee with a simple and chemical-free (solvent) sample preparation method [27,28,29,30,31]. In this present work, we use a chemical-free (no solvent used) sample preparation for honey authentication. To the best of our knowledge, there is no report of authentication of Indonesian honey according to their botanical, entomological and geographical origins by UV spectroscopy. Therefore, our objective is to implement a simple, inexpensive, and chemical-free analytical method based on UV spectroscopy and SIMCA (soft independent modeling of class analogy) for the classification of Indonesian honey according to its botanical, entomological, and geographical origins.

## 2. Materials and Methods

### 2.1. Honey Samples from Different Botanical, Entomological, and Geographical Origins

**Figure 1:**
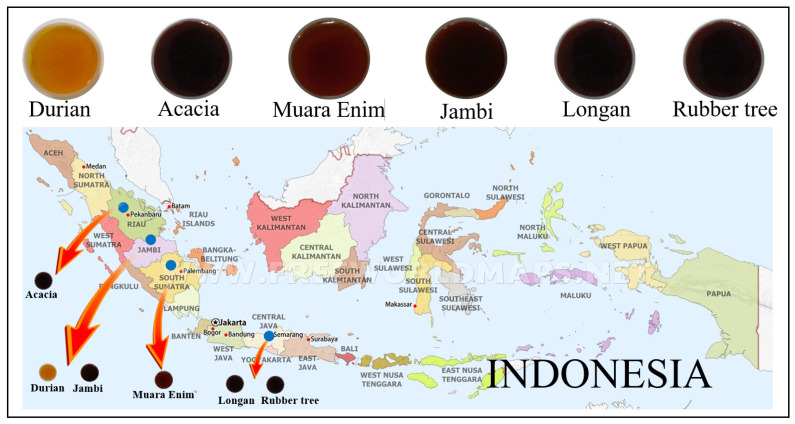
Geographical origin of Indonesian honeys used in this study.

Four types of honey collected by *Apis dorsata* bees were used: monofloral acacia (*Acacia mangium*), monofloral durian (*Durio zibethinus*), multifloral Muara Enim, and multifloral Jambi honey. Two types of honey collected by *Apis mellifera* bees were also used: monofloral longan (*Euphorbia longan*) and monofloral rubber tree (*Hevea brasiliensis*) honey. The samples were harvested during 2018 and 2019 from different origins in Indonesia (Table 1 and Figure 1). *Apis mellifera* honey from rubber tree and longan samples were harvested from a relatively homogeneous plantation of *Hevea brasiliensis* and *Euphorbia longan* in Batang, Central Java, Indonesia. All *Apis dorsata* honey samples were collected from a forest in Sumatra island, Indonesia. In Sumatra, there are five types of forest bioregion: protected forest, conservation forest, limited production forest, permanent production forest, and conversion forest. Monofloral acacia in Riau was harvested from a limited production forest bioregion with predominant vegetation of *Acacia mangium*, *Acacia crassicarpa*, and *Eucalyptus sp*. In Jambi and Muara Enim, multifloral honey samples were collected from the protected forest with various large plants such as *Koompassia excelsa*, *Bouea macrophylla Griffith*, *Lansium parasiticum,* and several small plants such as *Imperata cylindrica*. Monofloral durian honey from Jambi was harvested from a conversion forest. In this forest, farmers planted several fruit plants such as durian (*Durio zibethinus*), oil palm (*Elaeis guineensis*), and longan (*Euphorbia longan*), and woody plants such as *Hevea brasiliensis* and *Tectona grandis L.f*. A total of 2.5 kg of honey (non-filtered) was obtained for each type of honey and kept in plastic bottles at room temperature (20–25 °C) until analysis. The total number of samples of each type of honey was 120 for longan and rubber tree, and 200 for acacia, durian, Muara Enim, and Jambi; a total of 1040 honey samples were collected for analysis. These samples were randomly divided into three sample sets, namely calibration (524 samples), validation (344 samples), and prediction (172 samples).

### 2.2. UV Spectra Data Acquisition

The collected honey samples crystallized during storage in the laboratory. Prior to spectral measurement, honey samples were heated using a water bath at 60 °C for 30 min to liquefy the crystallized honey and obtain a homogenized honey sample, and were then kept at room temperature [32]. To take UV spectral measurements each honey sample was diluted 1:20 (mL:mL) using distilled water. A total of 2 mL of the diluted honey was pipetted into a 10 mm quartz cuvette. The UV spectra (190–400 nm) at 1 nm intervals of the honey samples were obtained using a low-cost benchtop UV-Vis spectrometer (Genesys™ 10S UV-Vis, Thermo Scientific, Waltham, MA, USA) in transmittance mode. The spectral acquisition was performed at room temperature. Three different pre-processing algorithms, namely 11 points of moving average smoothing (MAS), mean normalization (MN), and Savitzky–Golay first derivative with 11 points, and second-order polynomial fitting (ordo 2) (SG 1d) were simultaneously used in sequence to improve the obtained raw spectral data.

### 2.3. Chemometrics Analysis

To explore the spectral data, PCA (principal component analysis) and SIMCA (soft independent modeling of class analogy) were used for chemometric analysis. PCA is an unsupervised pattern recognition technique, which provides results about similarities and differences between samples without knowing anything about them [33]. In general, PCA is used to decompose highly correlated and complex spectral data by projecting variables into simpler and fewer uncorrelated new variables called principal components (PCs). For each PC, the PCA calculated its score and loading. In most reported papers, the score plot of the first two PCs (PC1 × PC2) or the first three PCs (PC1 × PC2 × PC3) are used to visualize the cluster formation of the samples and investigate the possible occurrence of outliers. Moreover, the plot of loadings visualizes the contribution of PCs and determines important variables. Further explanation of the concepts and details of PCA have been described in previous reports [34,35].

SIMCA is a supervised pattern recognition technique and belongs to a family of modeling classifiers that distinguishes between members and non-members of different classes [36]. SIMCA enables samples to be classified into existing classes or groups, assigning new samples to one class, more than one class, or no class according to similarities with these classes. First, a PCA model was developed for each class in the calibration sample set. The developed SIMCA model was then validated using a leave-one-out cross-validation method for the validation sample set. The prediction sample set (unknown samples) was then compared to the class models and its membership assigned to classes according to two criteria: the distance from the model center (leverage) and the distance to the model (residual). The sample-to-model distance (Si) is a measure of how far the sample lies from the modeled class. It is computed as the square root of the sample residual variance. SIMCA results can be visualized in a Cooman’s plot (Si vs. Si) and confusion matrix. A Cooman’s plot typically has four quadrants (Q1–Q4) and each predicted sample is assigned to one of the four possible quadrants: Q1 is the upper left rectangle (for samples that belong to model class 1), Q2 is the lower right rectangle (for samples that belong to model class 2), Q3 is the lower-left rectangle (for samples that belong to both model classes 1 and 2), and Q4 is the upper-right rectangle (for samples that do not belong to either model class 1 or 2).

The sensitivity, specificity, and accuracy were calculated using the equations according to Xu et al. [37].

The Unscrambler X version 10.4 (64-bit) (Camo Software AS, Oslo, Norway) was used to perform spectra pre-processing, PCA, and SIMCA calculations.

## 3. Results and Discussion

### 3.1. Analysis of UV Spectra

**Figure 2:**
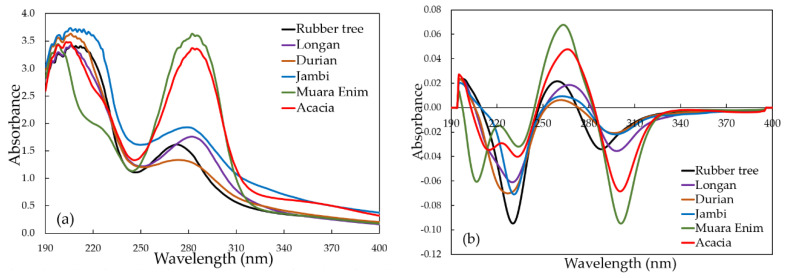
The average original (**a**) and pre-processed (**b**) spectra of the Indonesian honey with different botanical, entomological, and geographical origins over the range of 190–400 nm.

Figure 2 shows the averaged original or raw (a) and pre-processed spectra (b) of Indonesian honey of different botanical, entomological, and geographical origins. As it can be seen in Figure 2, in the raw UV spectra of 6 types of honey samples, large variations in the absorbance spectra were observed among different types of flora/botanical (monofloral versus multifloral), among the different geographical origins of the honey samples (Sumatra versus Java) as well as the different types of honeybees (*Apis dorsata* versus *Apis mellifera*). It was difficult to directly extract significant information from the raw spectra. For this reason, we improved the quality of the raw spectral data by applying spectral data pre-processing. Mean-normalization (MN) was performed as one of the spectral data pre-processing methods in this study. As was mentioned by Xing et al. [38], mean-normalization is one of the most classical normalization methods. It is equivalent to replacing the raw absorbance values by a profile centered on unity: only the relative absorbance values are used to describe the sample, and the information carried by their absolute levels is dropped. Savitzky–Golay first derivative with a second-order polynomial and a window size of 11 points (SG 1d) was used to cancel the baseline drifts and to enhance small spectral differences [39]. Due to similarity in honeybees (entomological), and geographical and botanical origins, especially for *Apis dorsata* multifloral from Jambi and *Apis dorsata* monofloral from Jambi, it was expected that the spectral differences within those honey samples were small. This is the main reason to use SG 1d—to enhance those small spectral differences. However, at the same time, as a consequence of derivation, the noises were also enhanced. To avoid this, the spectra were first smoothed using 11 points of moving averaging smoothing pre-processing (MAS) as recommended by previous work [39]. Therefore, in this present study, we utilized three sequential spectral data pre-processing methods: MAS, MN, and SG 1d (MAS + MN + SG 1d). A similar approach was previously used by Zhang et al. [39] and Shawky and Selim [40]. Overall, the shape of the spectral curves was quite similar, especially between 250–400 nm, with sharp differences in peak absorbance intensity at 270 and 300 nm. The two distinctive peaks observed at around 270 nm and 300 nm are associated with the absorbance of benzoic, salicylic, and aryl-alyphatic acids in honey [41]. These spectral results are consistent with previously reported work. Previously, UV–Vis absorption spectra of sixteen bulk Tuscany honey samples were reported, including acacia, clover, etc. [42]. Distinct peak absorbance intensities were observed around 270–280 nm depending on the type of honey. Minor peaks were also observed between 300–335 nm. The typical feature of original or raw UV spectral data is high noise with a very high absorbance (more than 2) especially in the interval of 190–250 nm (high-frequency noise). This raw spectral data is rich in unrelated information such as background information and systematic noise coming from the influences of light scattering, differences in path length, sample particle size, low lamp intensity at the start of spectral acquisition, and other factors [38]. Therefore, to achieve an acceptable result, in this present study for further chemometrics calculation we utilized relatively low noise spectral data using pre-processed spectral data in the interval of 250–400 nm.

### 3.2. PCA Analysis

**Figure 3:**
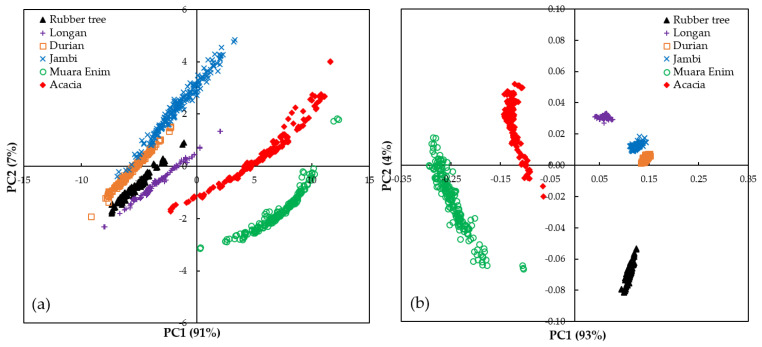
The score plot of the first two PCs (principal components; PC1 × PC2) for both the original (**a**) and pre-processed spectra (**b**) between 250–400 nm for the six different types of honey collected.

Figure 3 shows the results of PCA analysis in a two-dimensional score plot of the first two PCs (PC1 × PC2) in the original UV spectra (a) and pre-processed UV spectra of the honey samples (b). PCA was calculated using 1040 honey samples (including all spectra) from both the original and pre-processed spectral data (250–400 nm). The cumulative informative variance (CIV) for the two PCs was 98% and 97% for original and pre-processed spectra, respectively. This indicates that most of the variance in the original dataset was contained in these two principal components. While both the original and pre-processed spectra could be used to separate the honey samples, when using the original spectra some overlapping samples were observed between the clusters, especially in the PC1 direction along the *x*-axis. For example, the durian cluster had a very similar PC1 to that of the Jambi cluster. Clearer separation was obtained using the pre-processed spectra. Even so, the durian and Jambi honey clusters were still very close to each other. Both these honeys were harvested from the same geographical origin (Jambi), as shown in Table 1. In the PC1 direction, which accounted for 93% of the explained variance, all clusters could be discriminated with no overlapping samples being observed. It is evident that rubber tree, longan, durian, Jambi, Muara Enim, and acacia honey could be correctly classified. Therefore, further chemometrics analysis of SIMCA was performed using the pre-processed spectra (250–400 nm) to classify the Indonesian honey samples according to their botanical, entomological, and geographical origins.

**Figure 4:**
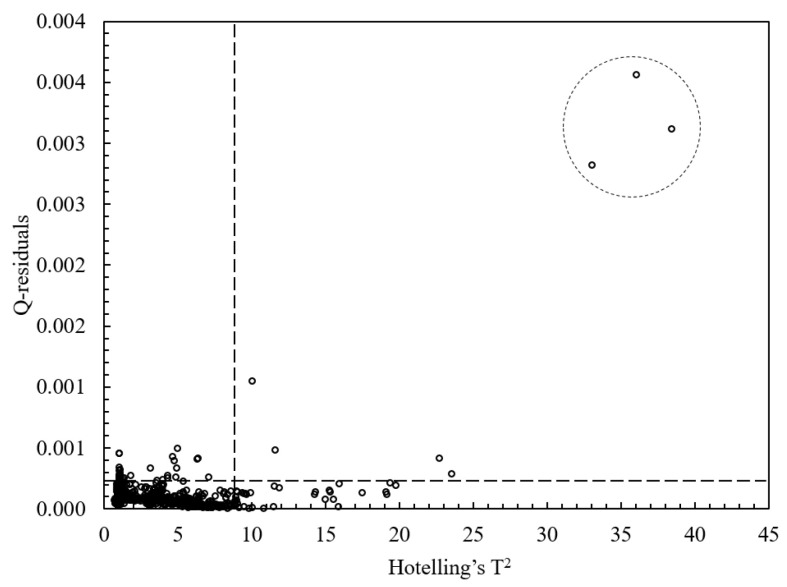
A plot of Hotelling’s T^2^ versus Q-residual of honey samples using the third principal component (PC3) from PCA (principal component analysis) calculation at the 95% significance level.

To detect the possible occurrence of outliers, a Hotelling’s T^2^ versus Q-residual from the PCA calculation at the 95% probability level was plotted (Figure 4) using the third principal component (PC3). Hotelling’s T^2^ is a common approach to determine the significance of multivariate distances, while Q-residual represents how well samples are in accordance with the model. Samples with both a high Q-residual and Hotelling’s T^2^ are more likely to be outliers. According to the Hotelling’s T^2^ and Q residuals tests, three Muara Enim samples were identified as outliers and removed before further analysis.

**Figure 5:**
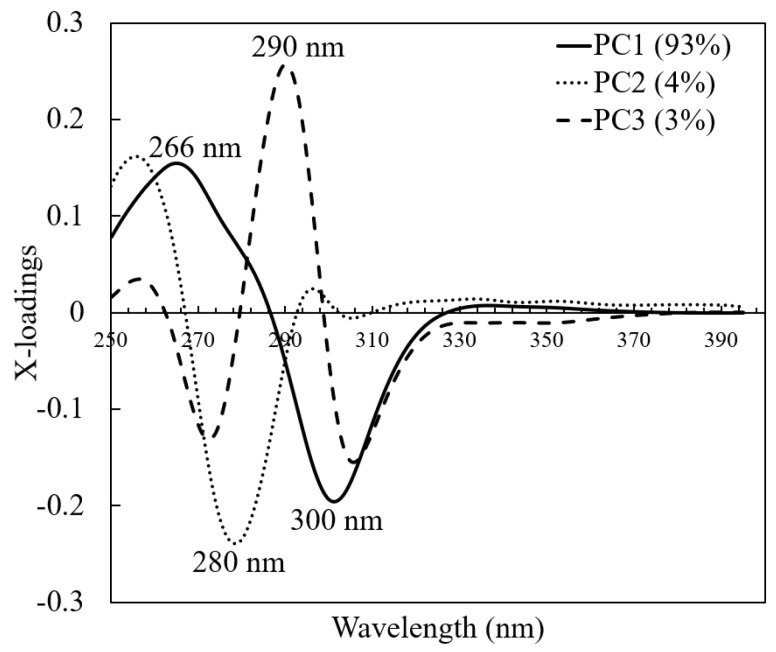
X-loadings plots for all three components for pre-processed spectra.

An x-loadings plot of the first three PCs of the pre-processed spectra was used to evaluate the most influential variables to classify honey samples (Figure 5). The x-loadings plot of the PC1, which accounts for 93% of the variance, has a positive peak at 266 nm and a negative peak at 300 nm. The PC2 and PC3, which account for 7% of the total variance, had a negative peak at 280 nm and a positive peak at 290 nm.

### 3.3. Supervised Classification of SIMCA

A SIMCA model for each class was created using calibration and validation samples as shown in Table 2 (total 868 samples for 6 classes). The optimum number of principal components (PCs) used for each class was determined by using a leave-one-out cross-validation method. As seen in Table 2, a SIMCA model for each class was constructed with a different number of optimum PCs. Three PCs were used to construct class rubber tree, longan, Jambi and acacia SIMCA models with the obtained CIV in calibrations of 99.489, 99.520, 99.113, and 99.059%, respectively. Two PCs were used to develop class durian and Muara Enim SIMCA models with the obtained CIV in calibration of 98.054 and 98.667%, respectively.

To evaluate the classification performance of the analyses investigated between all the different honey samples collected in terms of botanical, entomological, and geographical origins, we considered six classes of honey: rubber tree, longan, durian, Jambi, Muara Enim, and acacia. The developed SIMCA models were used to verify the membership of the prediction sample sets into six available classes. The prediction results are presented in Table 3. According to Xu et al. [37], the accuracy, sensitivity, and specificity were 100% for all classes.

**Figure 6:**
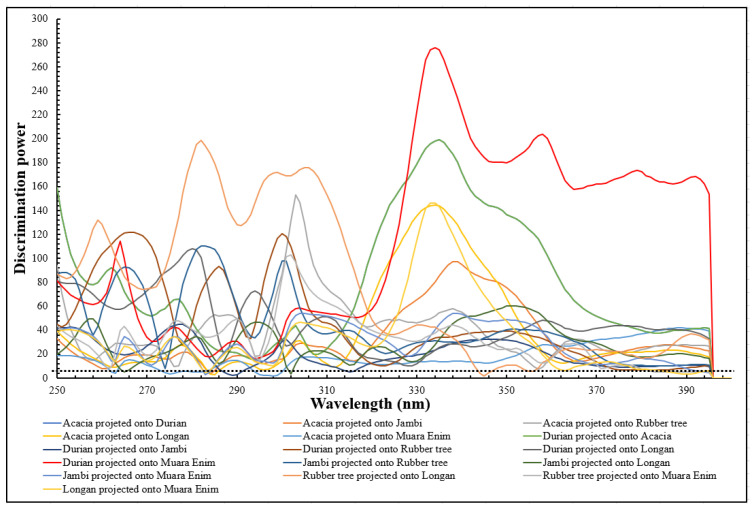
The discrimination power plot of wavelengths obtained by prediction sample sets using pre-processed spectra over the range of 250–400 nm (the black dashed line at discrimination power = 3 is a threshold for selecting important wavelengths).

The model distances for the six classes obtained by the SIMCA classification are presented in Table 4. These values are usually utilized to estimate the distance between the models to quantify possible differences between the models. It should be noted that a model distance and discrimination power (dp) larger than 3 indicates good class separation and that the models are significantly different with a low risk of misclassification in the model [43,44,45]. As can be seen in Table 4, model distances were larger than 3 for all classes, indicating that the developed SIMCA models were significantly different between the six honey types collected. To evaluate the most influential wavelength for discriminating between the models, discrimination power values of the SIMCA models were also plotted against wavelength (Figure 6). For the spectra data from 250–400 nm, the discrimination power was greater than 3. This confirms the spectral window selected (250–400 nm) was appropriate for classifying the different types of Indonesian honey collected. Specific wavelengths had a very high discrimination power: at 270 nm (dp = 120), 280 nm (dp = 200), 300 nm (dp = 175), 335 nm (dp = 275), and 360 nm (dp = 200). These wavelengths are extremely important in the discrimination of the types of Indonesian honey collected.

**Figure 7:**
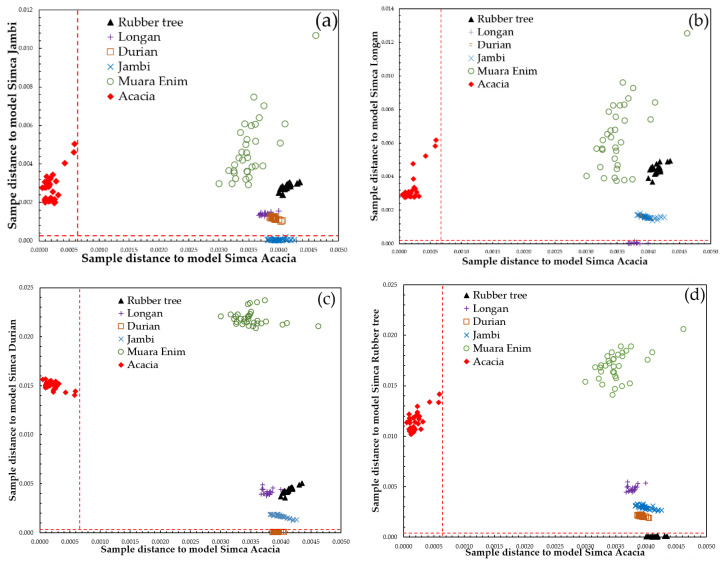

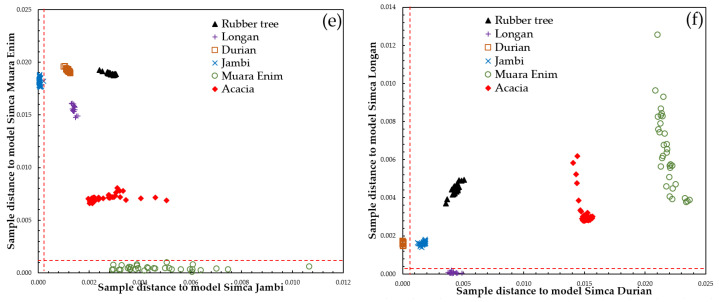
Cooman’s plot of the classification result of six pairwise SIMCA models for prediction sample sets using SIMCA models developed in each class using pre-processed spectral data over the range of 250–400 nm (acacia versus Jambi (**a**), acacia versus longan (**b**), acacia versus durian (**c**), acacia versus rubber tree (**d**), Jambi versus Muara Enim (**e**), and longan versus durian (**f**)). (the significance level for the Cooman’s plot is 95%). (Rubber tree: black triangle; longan: purple plus; durian: brown open squares; Jambi: blue cross; Muara Enim: green open circle; acacia: red diamonds).

SIMCA prediction results are also presented in the form of a Cooman’s plot (Figure 7). There are six pairwise classes to be tested. The Cooman’s plots for these are shown in Figure 7: acacia versus Jambi (a), acacia versus longan (b), acacia versus durian (c), acacia versus rubber tree (d), Jambi versus Muara Enim (e), and longan versus durian (f). The red dashed lines in Cooman’s plot are membership lines with a 95% confidence limit that divide the membership area into four quadrants. In Figure 7a, the *x*-axis and *y*-axis denote the distance to acacia and Jambi samples. All acacia samples appeared in the upper left quadrant and were properly classified into the acacia class (33 samples). The Jambi samples were located in the lower right quadrant (32 samples), which indicates correct classification into the Jambi class, with only one Jambi sample falling in the upper right quadrant (belong to neither class). However, no samples were plotted in the lower left quadrant (belongs to both classes). Rubber tree (20 samples), durian (33 samples), Muara Enim (33 samples), and longan (20 samples) were successfully rejected as belonging either to the acacia or Jambi classes and were plotted in the upper right quadrant. Similar results were observed for other pairwise classes, as can be seen in Figure 7b–f. This demonstrates the capability of the developed SIMCA model to classify Indonesian honeys according to their botanical, entomological, and geographical origins.

In this research, a satisfactory classification of the different types of Indonesian honey collected from different botanical, entomological, and geographical origins was achieved using pre-processed UV spectral data (250–400 nm). Supervised classification using SIMCA reported an accuracy, sensitivity, and specificity of 100%. Several specific wavelengths were identified for accurate classification of Indonesian honey types, including 266, 270, 280, 290, 300, 335, and 360 nm. It should be noted that the obtained spectra were consistent with the results reported in previous studies. A spectral window between 200–400 nm provided the means to accurately classify and distinguish between monofloral genuine Sidr honey from different geographical origins, the season of harvest, and non-Sidr honey. Using HCA and PCA, obvious grouping patterns between the genuine Sidr and non-Sidr honeys were achieved [26]. However, there was no further explanation on specific wavelengths that were responsible for the discrimination. Using fluorescence spectroscopy, an excitation wavelength with a spectral window between 260–290 nm had strong emission spectra between 330–360 nm. Fluorescence spectra in this region originate from the aromatic amino acids and are commonly used for the determination of the botanical origin of honey [46,47]. The excitation at 360 nm was associated with the fluorescent Maillard reaction products present in honey, such as furosine and hydroxymethylfurfural, and exhibited emission peaks at 440 and 425 nm [47]. In another study, excitation at 280 nm was used to classify raw Ethiopian honeys using front-face fluorescence spectroscopy [48]. Recently, excitations at 270–290 nm and 320–340 nm were also reported to be appropriate for detecting emission spectra of minor components in honey such as amino acids (mainly tryptophan), proteins, and some phenolic acids [42].

## 4. Conclusions

In this study, PCA and SIMCA were performed on UV spectral data from Indonesian honey samples of different botanical, entomological, and geographical origins. The subsequent evaluation of these models demonstrated that this UV spectroscopy along with chemometrics can be used as a simple, chemical-free (no toxic waste), and low-cost analytical method for the authentication of Indonesian honeys from differing botanical, entomological, and geographical origins. PCA of the spectra, together with SIMCA, confirmed that the different honey types could be distinguished based on specific, sensitive wavelengths within the 200 to 400 nm range, and potential outliers were detected. The prediction performance of these developed models had an accuracy, sensitivity, and specificity of 100% for all models. Several specific wavelengths were identified as being highly sensitive and specific fingerprints of the Indonesian honey types were investigated. These wavelengths are closely associated with the optical properties of important chemical components in these honeys. The present study provides a foundation for developing robust models for distinguishing between the botanical, entomological, and geographical origins of Indonesian honey types, as well as between different harvest years.

## Figures and Tables

**Table 1:** The characteristics of the honey samples and their origin.

Honey Sample	Floral Types	Floral Sources	Bee Types	Geographical Origin	Number of Samples
Rubber tree	Monofloral	*Hevea brasiliensis*	*Apis mellifera*	Central Java	120
Longan	Monofloral	*Euphorbia longan*	*Apis mellifera*	Central Java	120
Durian	Monofloral	*Durio zibethinus*	*Apis dorsata*	Jambi	200
Jambi	Multifloral	-	*Apis dorsata*	Jambi	200
Muara Enim	Multifloral	-	*Apis dorsata*	South Sumatera	200
Acacia	Monofloral	*Acacia mangium*	*Apis dorsata*	Riau	200

**Table 2:** The result of SIMCA (soft independent modeling of class analogy) model development for each class using calibration and validation sample sets.

SIMCA Model	Number of Calibration and Validation Samples	Number of Principal Components (PCs)	The Cumulative Informative Variance (CIV) (%)	
			Calibration	Validation
Rubber tree	100	3	99.489	98.893
Longan	100	3	99.520	98.962
Durian	167	2	98.054	98.155
Jambi	167	3	99.113	99.279
Muara Enim	167	2	98.667	98.910
Acacia	167	3	99.059	98.441

**Table 3:** Confusion matrix of for SIMCA prediction result using prediction sample sets.

		Predicted Classes					
		Rubber Tree	Longan	Durian	Jambi	Muara Enim	Acacia
Actual Classes	Rubber tree	18	0	0	0	0	0
	Longan	0	19	0	0	0	0
	Durian	0	0	33	0	0	0
	Jambi	0	0	0	31	0	0
	Muara Enim	0	0	0	0	32	0
	Acacia	0	0	0	0	0	32

**Table 4:** Model distance for each class calculated from prediction result using prediction sample sets.

		Model Distance					
		Rubber Tree	Longan	Durian	Jambi	Muara Enim	Acacia
Classes	Rubber tree	1	9139	4090	2783	2034	2403
	Longan		1	3774	802	915	427
	Durian			1	653	2573	3711
	Jambi				1	1121	386
	Muara Enim					1	167
	Acacia						1

## Data Availability

Data sharing is not applicable to this article.
